# Correction: Incomplete dominance of deleterious alleles contributes substantially to trait variation and heterosis in maize

**DOI:** 10.1371/journal.pgen.1009825

**Published:** 2021-09-28

**Authors:** Jinliang Yang, Sofiane Mezmouk, Andy Baumgarten, Edward S. Buckler, Katherine E. Guill, Michael D. McMullen, Rita H. Mumm, Jeffrey Ross-Ibarra

In Fig 1, panel a is incorrect. Please see the correct Fig 1 here.

**Fig 1:**
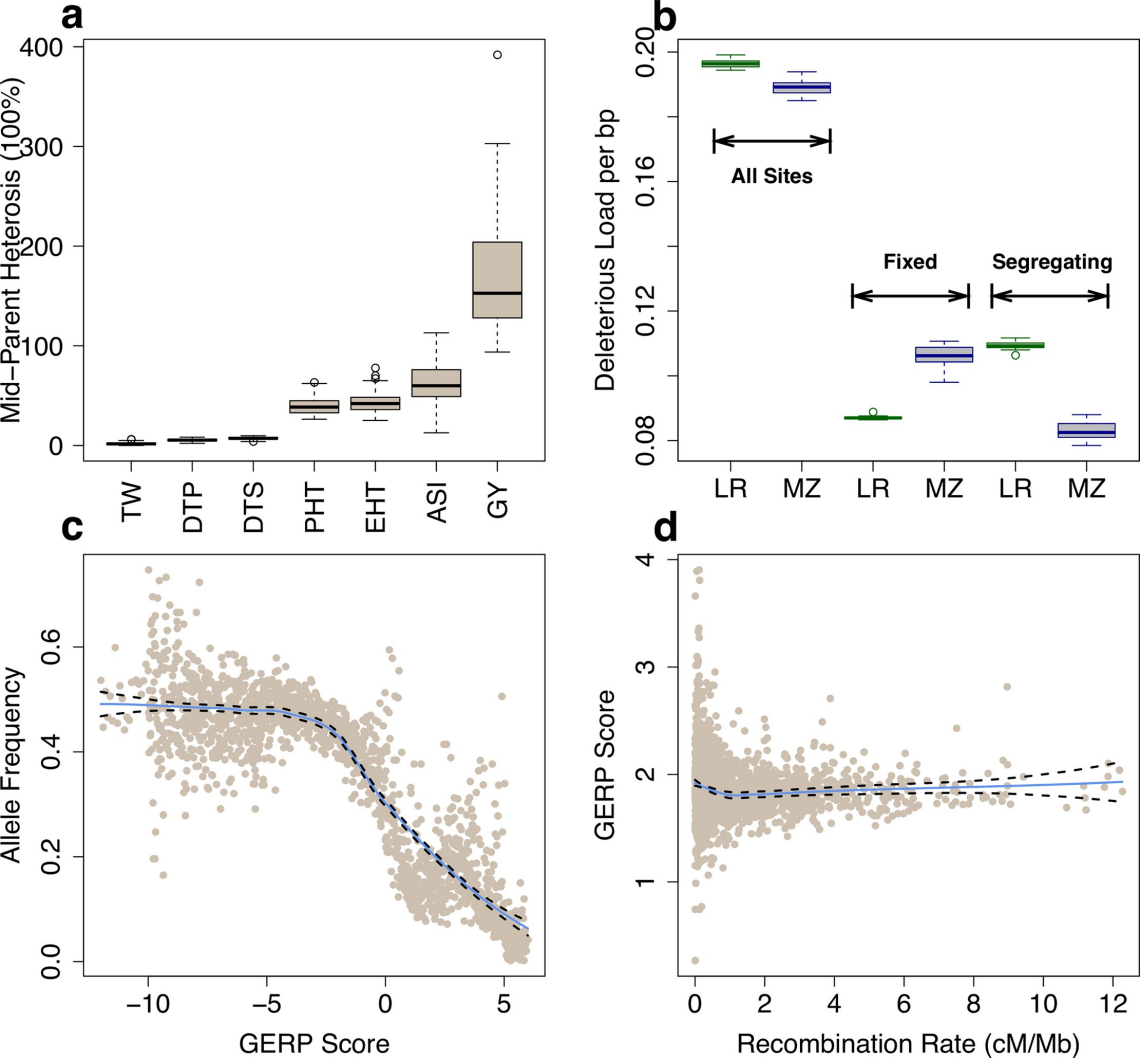
Heterosis and deleterious variants. (**a**) Boxplots (median and interquartile range) of percent mid-parent heterosis (MPH). (**b**) Proportion of deleterious alleles in landraces (LR, green) and elite maize (MZ, blue) lines. (**c**) The allele frequency of the minor alleles in the multi-species alignment in bins of 0.01 GERP score (including GERP < = 0 sites). (**d**) The mean GERP score for putatively deleterious sites (GERP >0). Each point represents a 1 Mb window. In (**c**) and (**d**) the solid blue and dashed black lines define the best-fit regression line and its 95% confidence interval.

In S1 Table, the values in the eleventh column, “Mid-parent_Heterosis”, are incorrect. Please view the correct S1 Table below.

## Supporting information

S1 Table Best Linear Unbiased Estimator (BLUE) values and levels of heterosis of the seven phenotypic traits for the 66 hybrids. Abbreviations for phenotypic traits are plant height (PHT, in cm), height of primary ear (EHT, in cm), days to 50% pollen shed (DTP), days to 50% silking (DTS), anthesis-silking interval (ASI, in days), grain yield adjusted to 15.5% moisture (GY, in bu/A), and test weight (TW, weight of 1 bushel of grain in pounds).
(CSV)
