# Supplementary figures and images for: Incomplete dominance of deleterious alleles contributes substantially to trait variation and heterosis in maize
Source: PLoS Genet. 2017 Sep 27;13(9):e1007019. doi: 10.1371/journal.pgen.1007019 (PMC5633198; doi:10.1371/journal.pgen.1007019)

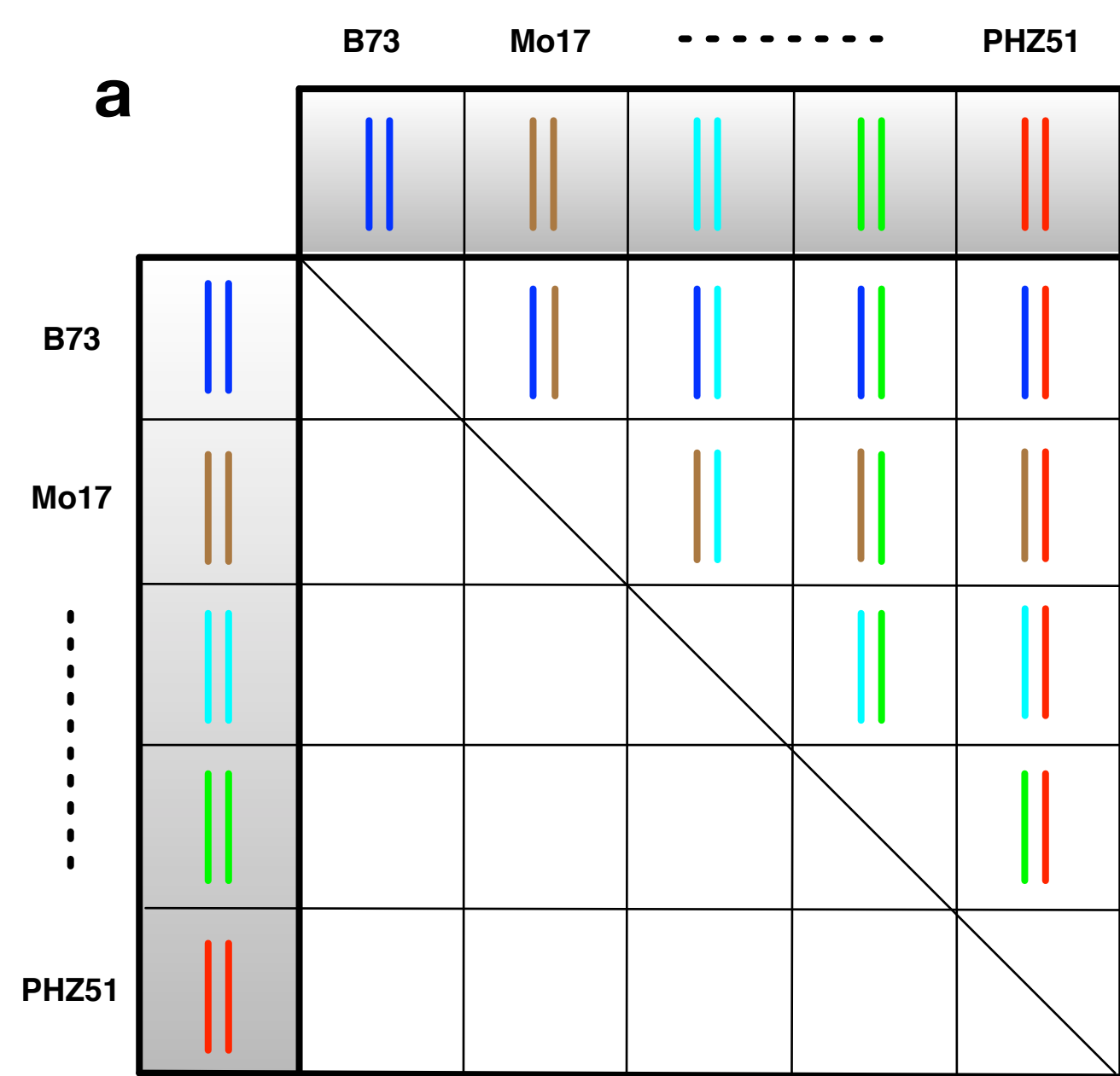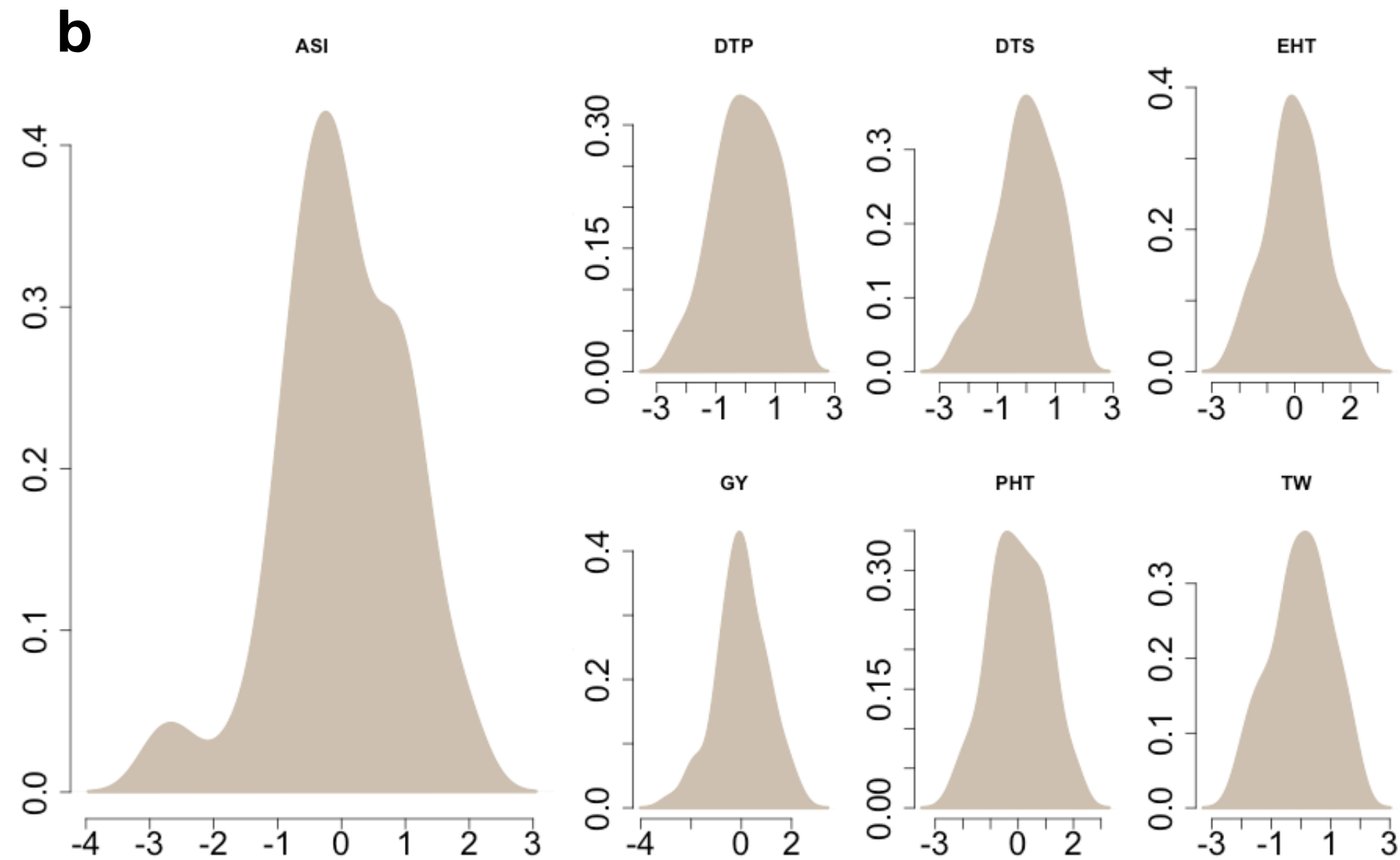

Supplement: S1 Fig — (a) Twelve maize inbred lines were selected and crossed in a half-diallel fashion. Each inbred lines was used as both male and female and the resulting F1 seed was bulked. (b) Density plots of normalized BLUE values for the seven phenotypic traits. We used the “scale” function in R to normalize the BLUE values by first centering on zero and then dividing the numbers by their standard deviation. The seven phenotypic traits are plant height (PHT), height of primary ear (EHT), days to 50% pollen shed (DTP), days to 50% silking (DTS), anthesis-silking interval (ASI), grain yield adjusted to 15.5% moisture (GY), and test weight (TW). (PDF) [file pgen.1007019.s001.pdf]

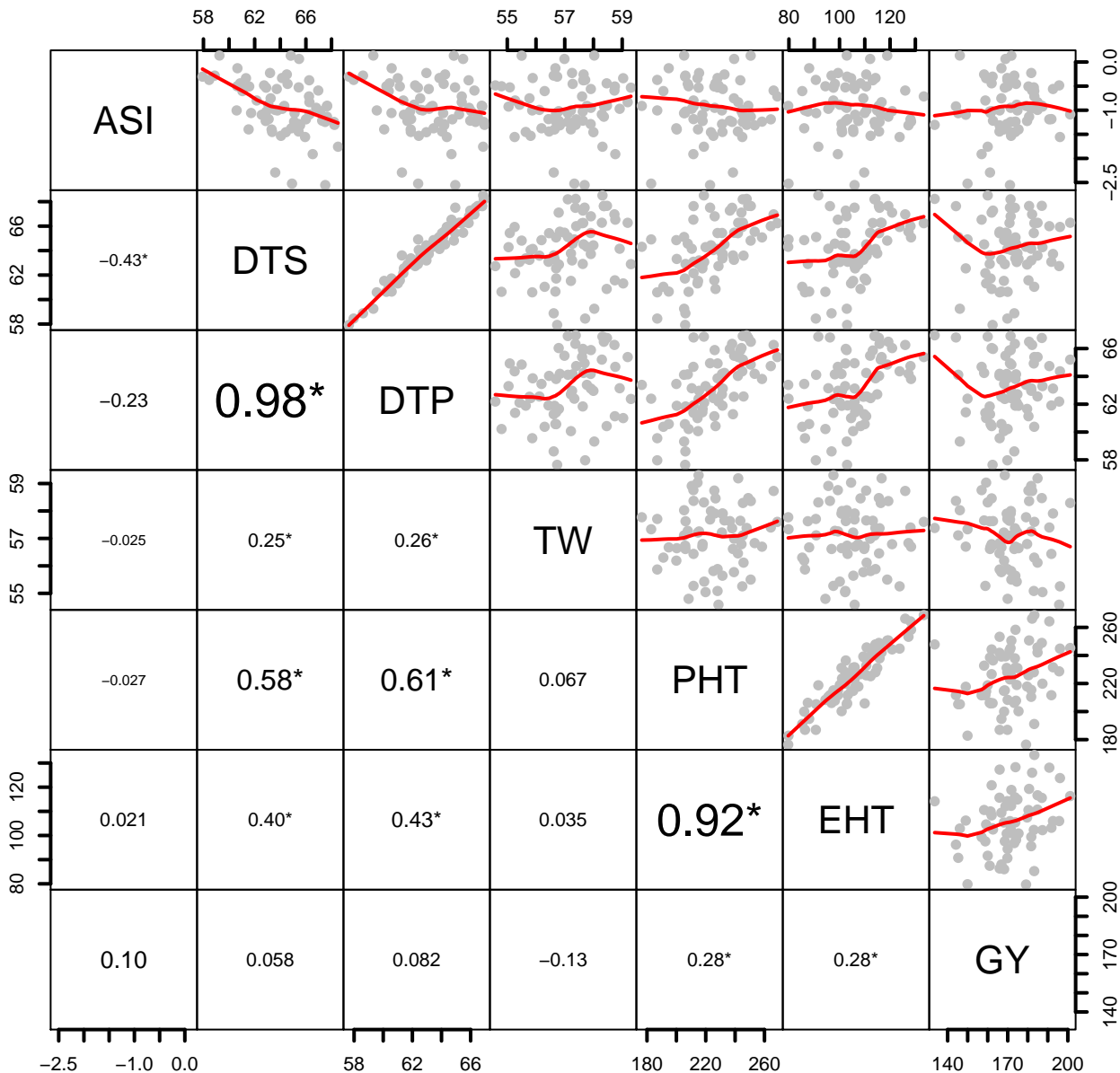

Supplement: S2 Fig — The upper right panels show the scatter plots of all possible pairwise comparisons of two traits. The red line is a fitted loess curve. In the lower left panels, the numbers are the Spearman correlation coefficients (r) and the asterisks (*) indicate the correlation coefficients are statistically significant (Spearman correlation test P value < 0.05). Units for various traits are plant height (PHT, in cm), height of primary ear (EHT, in cm), days to 50% pollen shed (DTP), days to 50% silking (DTS), anthesis-silking interval (ASI, in days), grain yield adjusted to 15.5% moisture (GY, in bu/A), and test weight (TW, weight of 1 bushel of grain in pounds). (PDF) [file pgen.1007019.s002.pdf]

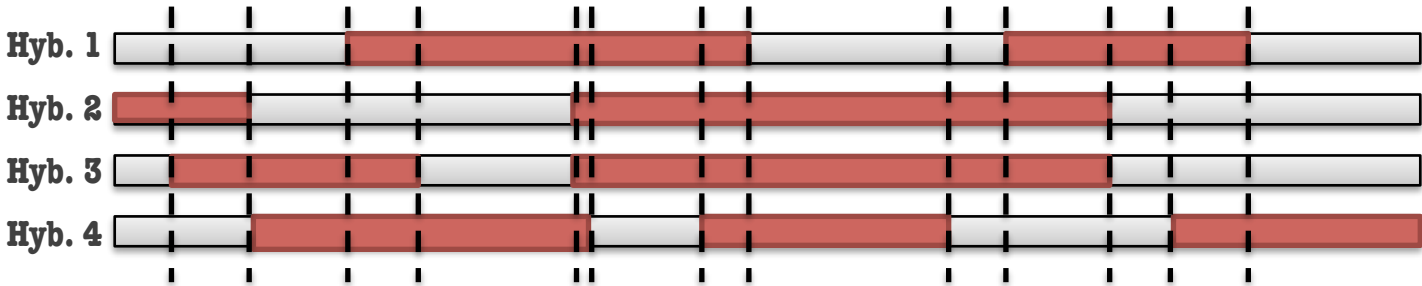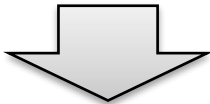

|      | HB1 | HB2 | HB3 | HB4 | HB5 | HB6 | HB7 | HB8 | HB9 | HB10 | HB11 | HB12 | HB13 | HB14 |
|------|-----|-----|-----|-----|-----|-----|-----|-----|-----|------|------|------|------|------|
| Hyb1 | 0   | 0   | 0   | 1   | 1   | 1   | 1   | 1   | 0   | 0    | 1    | 1    | 1    | 0    |
| Hyb2 | 1   | 1   | 0   | 0   | 0   | 1   | 1   | 1   | 1   | 1    | 1    | 0    | 0    | 0    |
| Hyb3 | 0   | 1   | 1   | 1   | 0   | 1   | 1   | 1   | 1   | 1    | 1    | 0    | 0    | 0    |
| Hyb4 | 0   | 0   | 1   | 1   | 1   | 1   | 0   | 1   | 1   | 0    | 0    | 0    | 1    | 1    |

Supplement: S3 Fig — In the upper panel, regions in red are IBD blocks identified by pairwise comparison of the two parental lines of a hybrid. The vertical dashed lines define haplotype blocks. In the lower panel, hybrid genotypes in each block are coded as heterozygotes (0) or homozygotes (1). (PDF) [file pgen.1007019.s003.pdf]

Avg. GERP

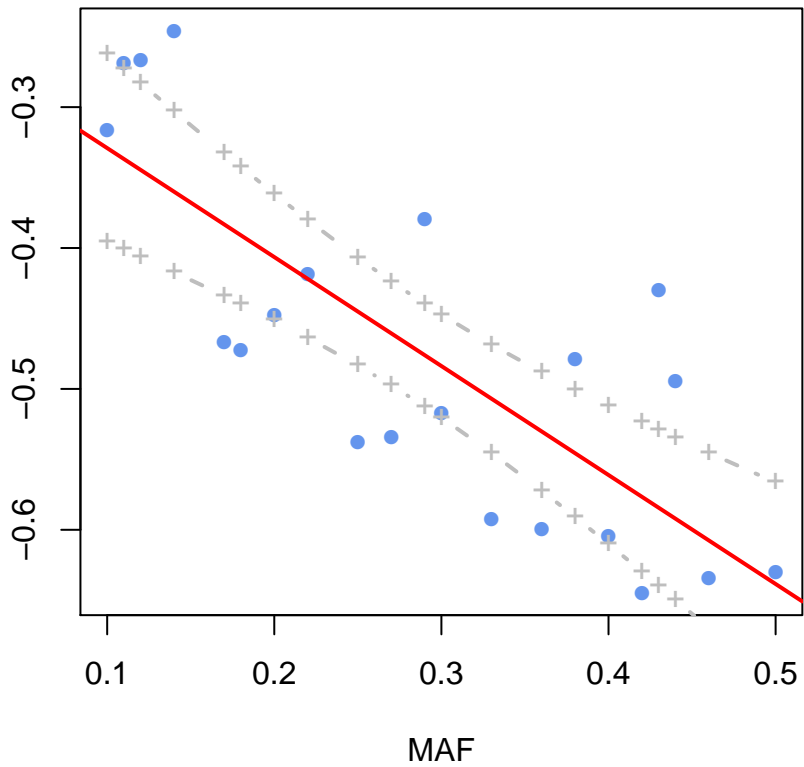

Supplement: S4 Fig — Red solid and grey dashed lines define the best-fit regression line and its 95% confidence interval. (PDF) [file pgen.1007019.s004.pdf]

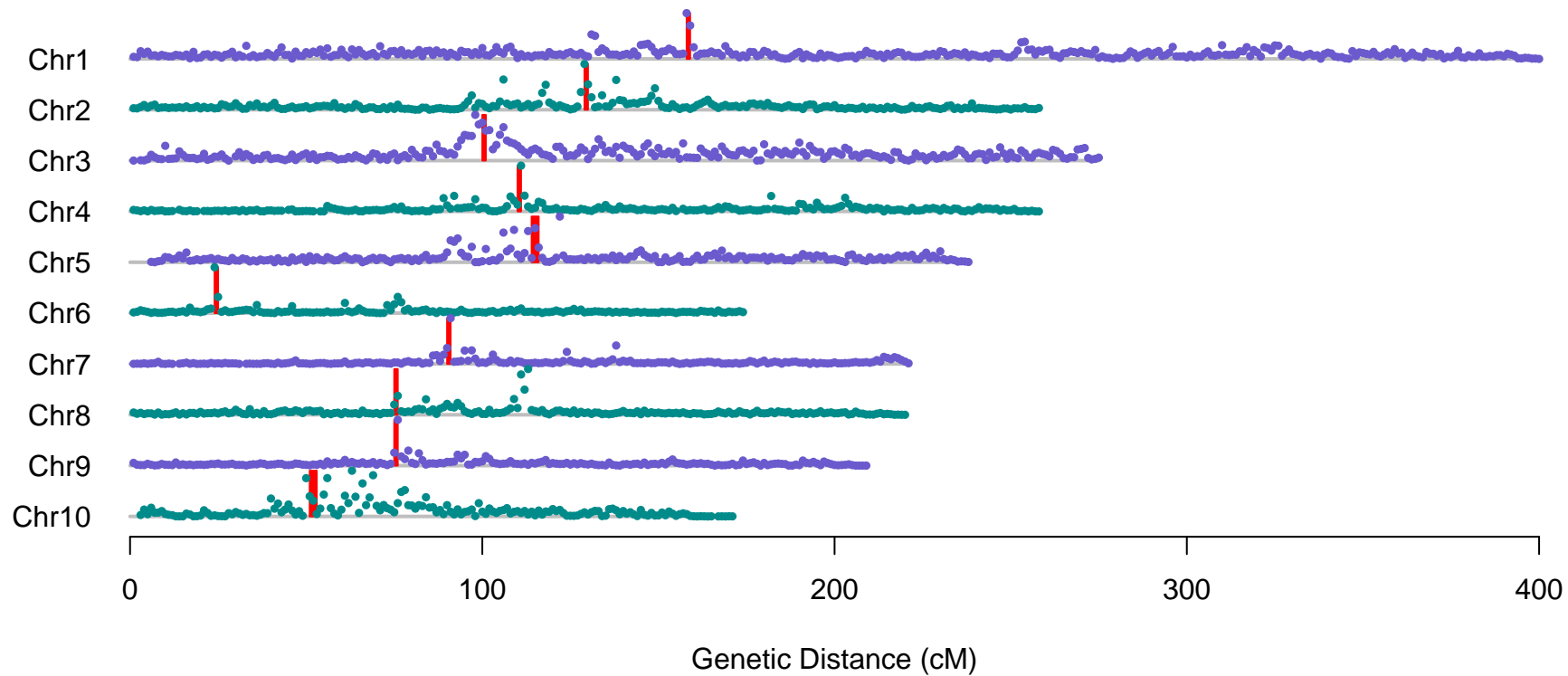

Supplement: S5 Fig — ots indicate mean GERP scores of putatively deleterious SNPs (GERP scores > 0) carried by the 12 parental maize lines (bin size = 1 cM). Vertical red lines indicate centromeres. (PDF) [file pgen.1007019.s005.pdf]

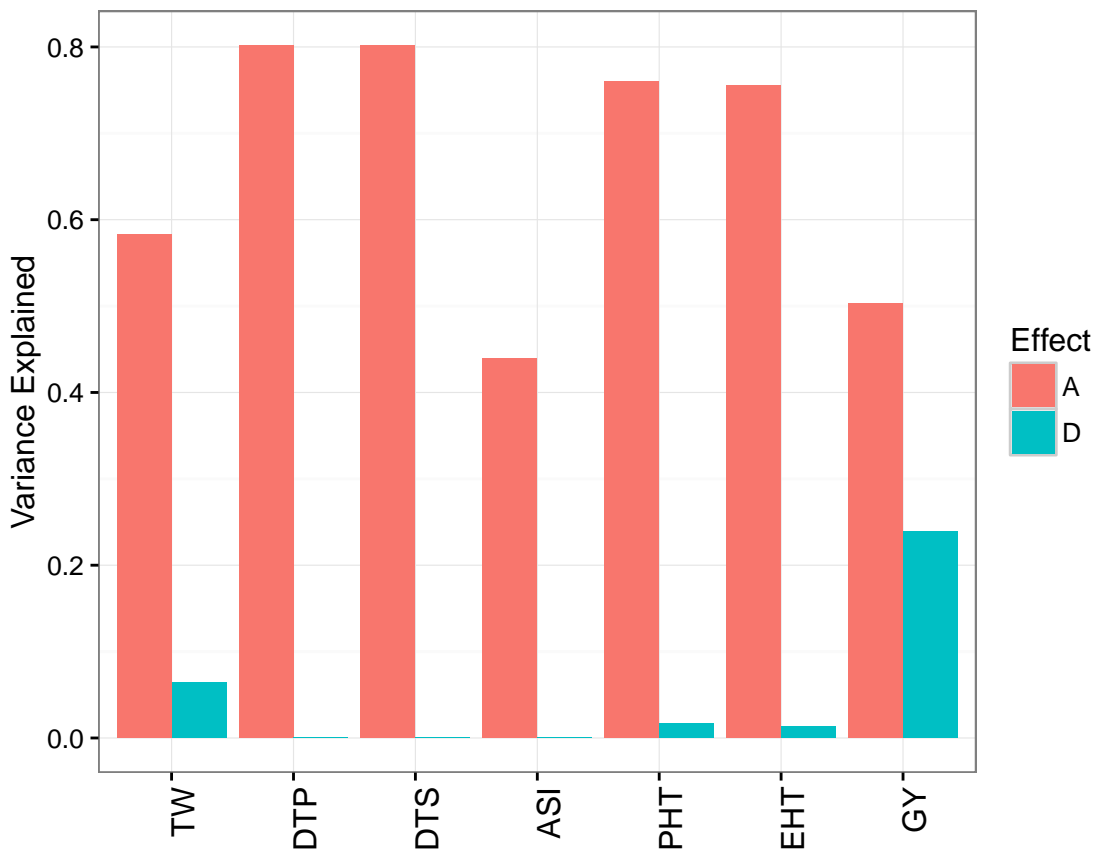

Supplement: S6 Fig — Additive and dominance effects are indicated by red and blue colors respectively. (PDF) [file pgen.1007019.s006.pdf]

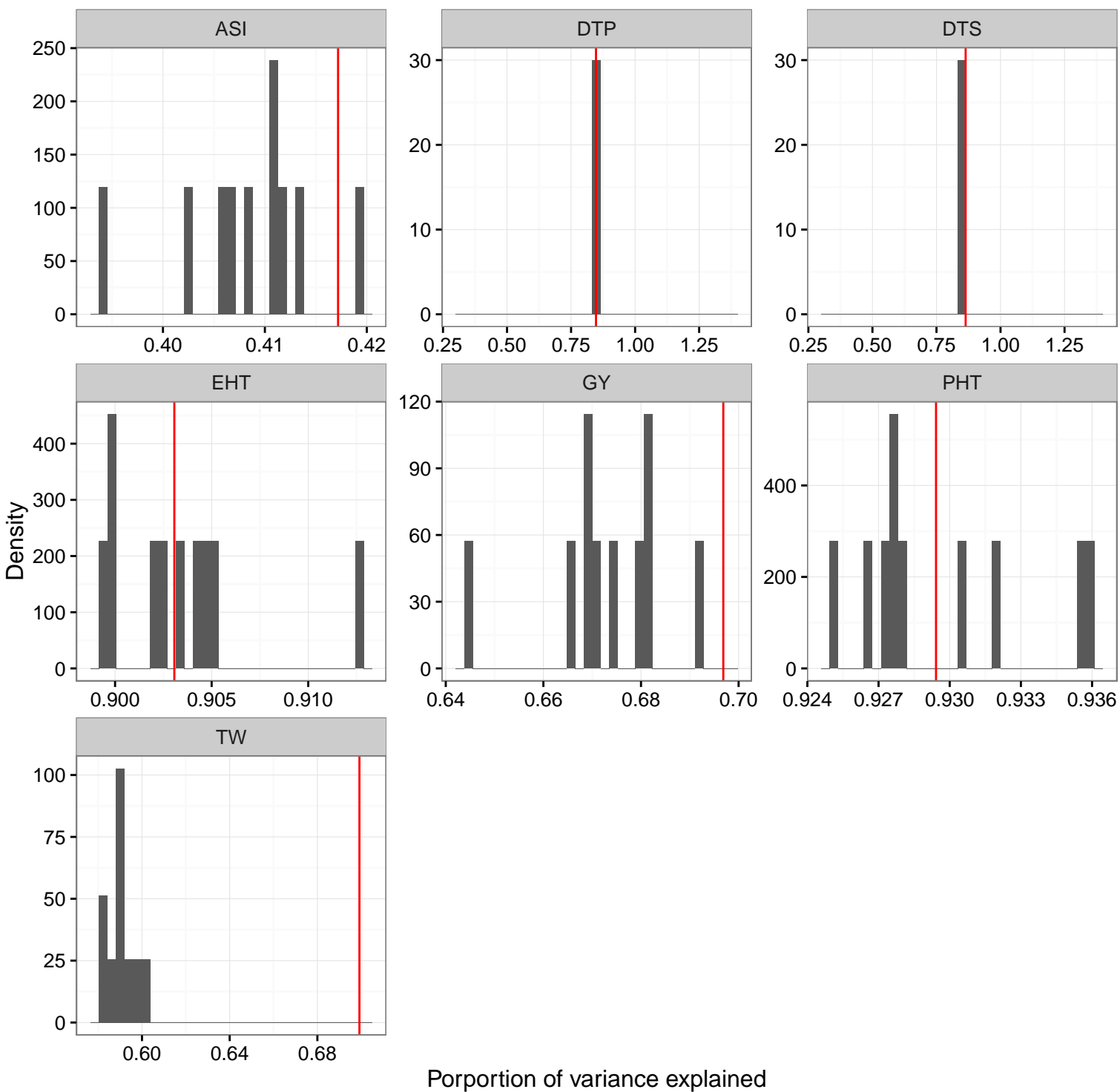

Supplement: S7 Fig — Histograms show the results for the randomly shuffled (10 times) degrees of dominance (k) in each trait. Red lines show the phenotypic variance explained using the observed k. (PDF) [file pgen.1007019.s007.pdf]

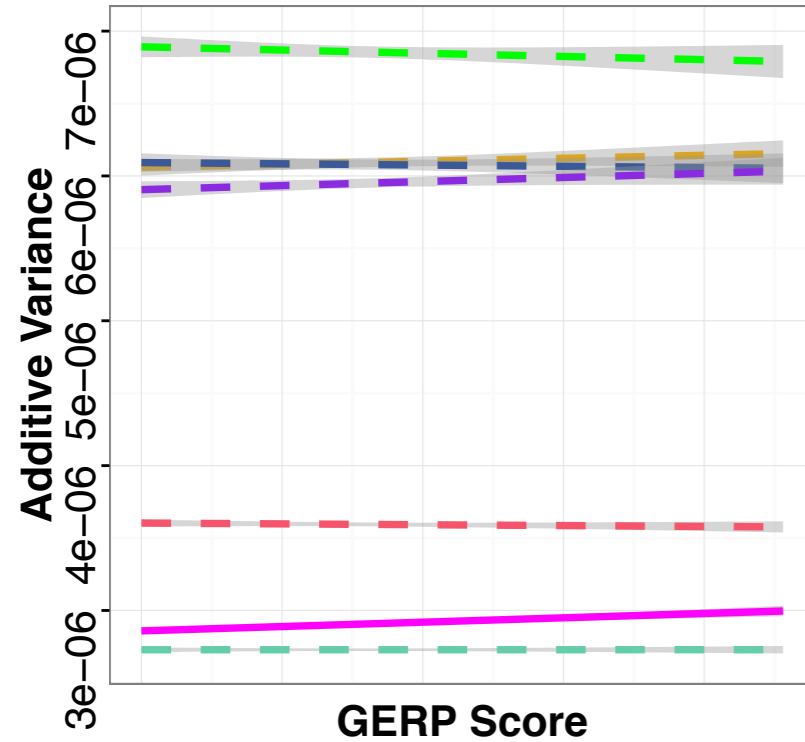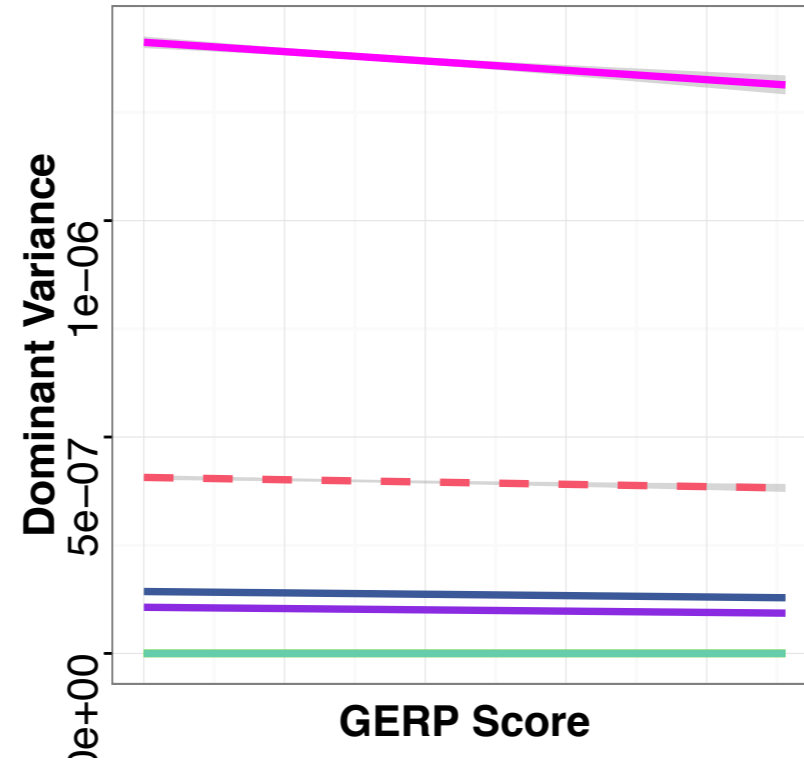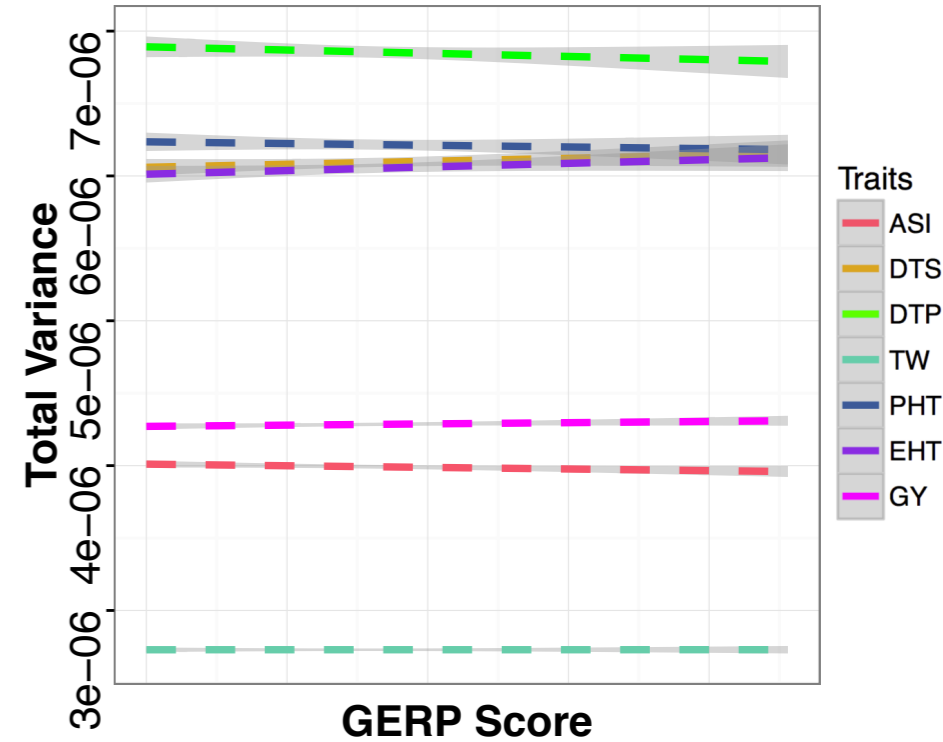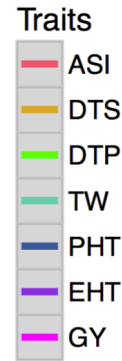

Supplement: S8 Fig — Solid and dashed lines represent significant and non-significant linear regressions, with grey bands representing 95% confidence intervals. Data are only shown for SNPs which explain more phenotypic variance than the genome-wide mean. (PDF) [file pgen.1007019.s008.pdf]

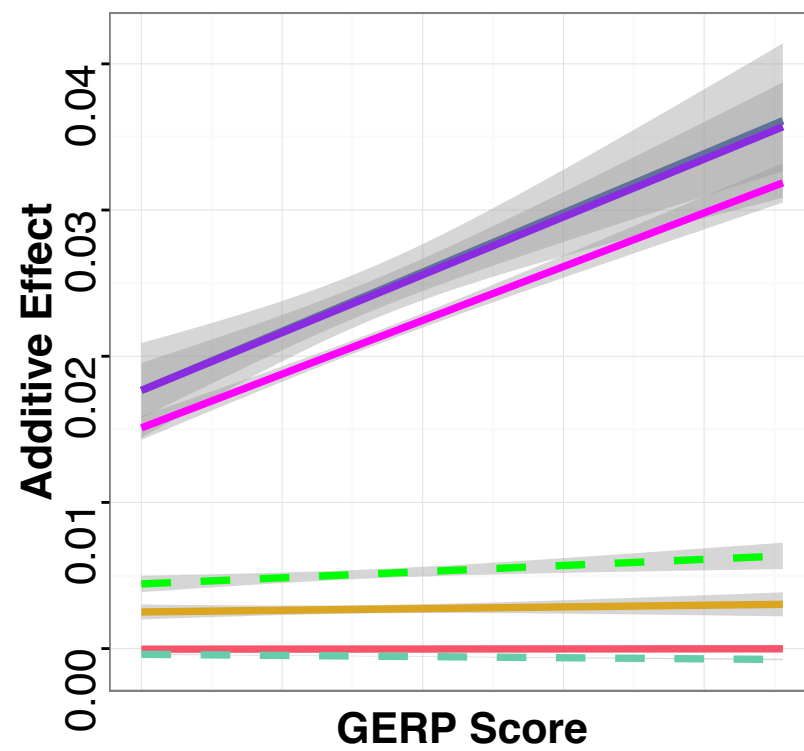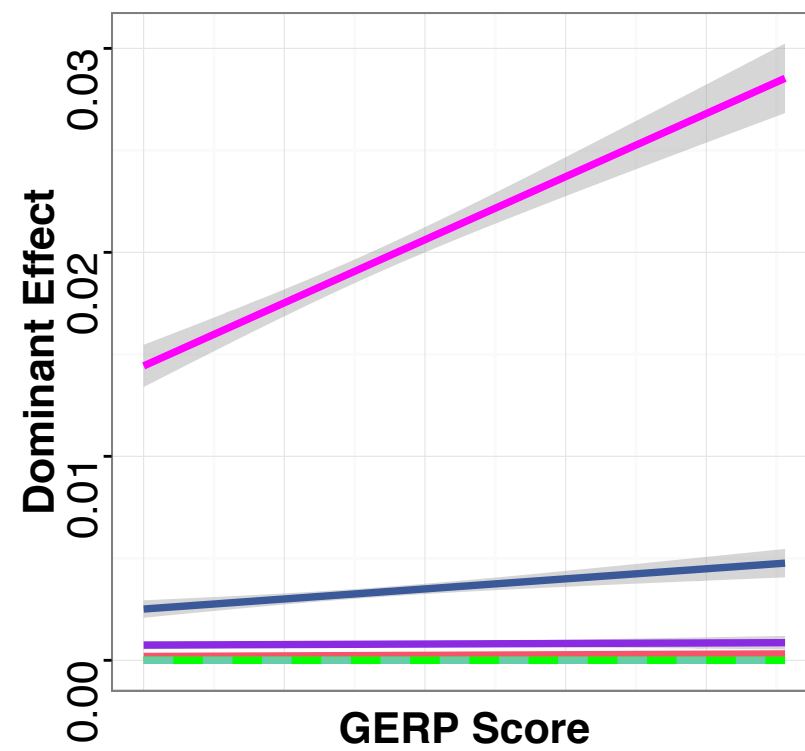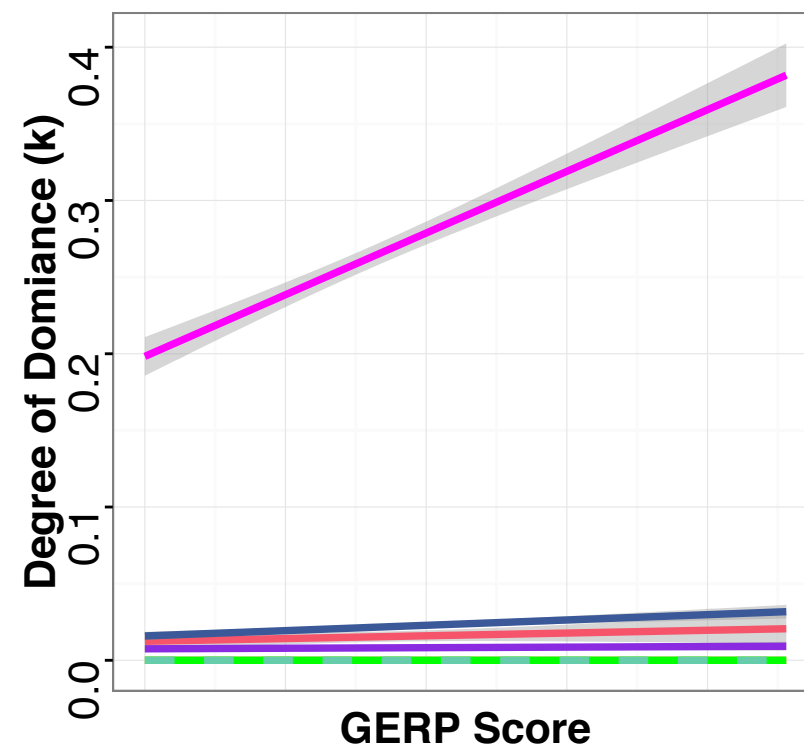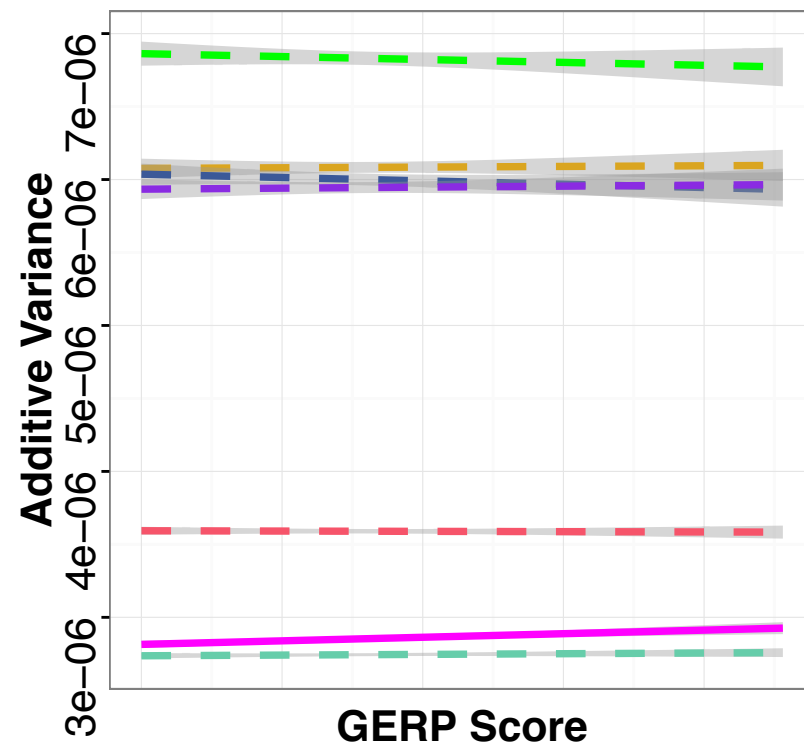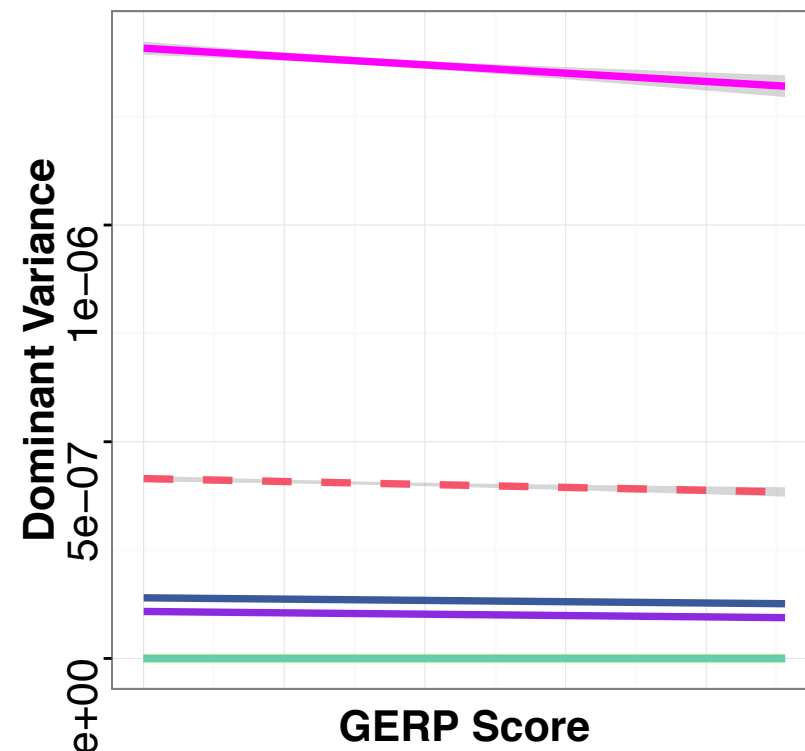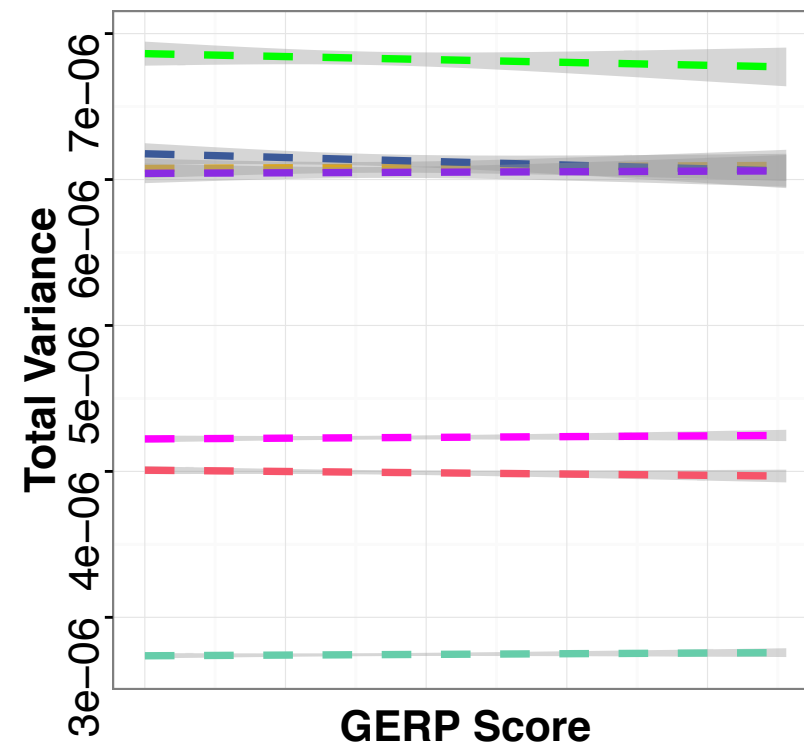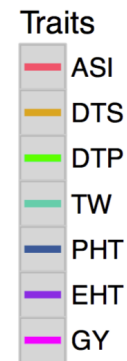

Supplement: S9 Fig — Solid and dashed lines represent significant and non-significant linear regressions, with grey bands representing 95% confidence intervals. Data are only shown for GERP-SNPs which explain more variance than the genome-wide mean and found in regions above the first quantile of the recombination rate (cM/Mb). (PDF) [file pgen.1007019.s009.pdf]

**a** Grain Yield excluding B73-related Hybrids

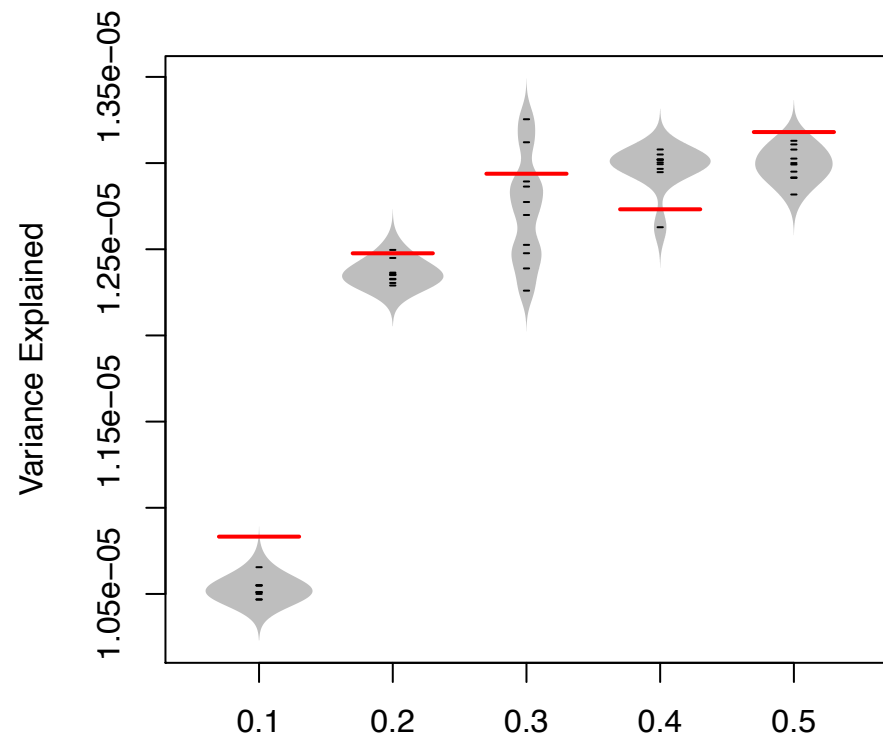

**b**

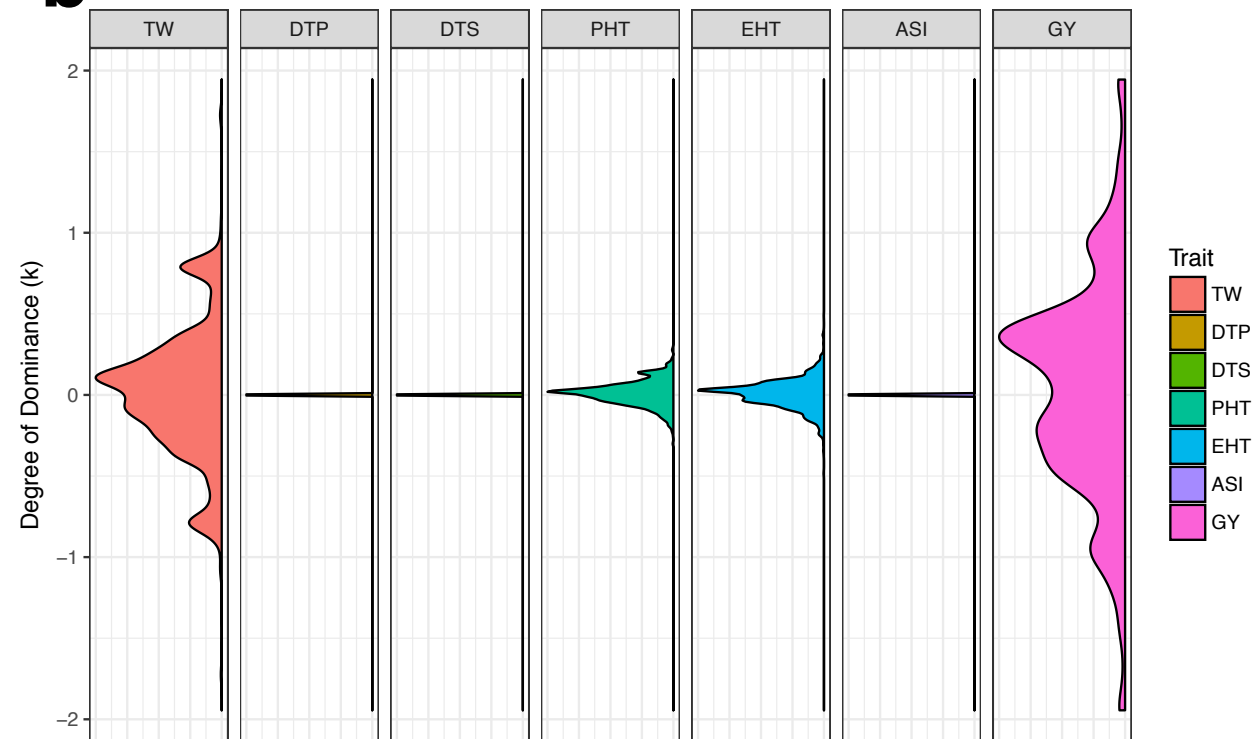

**c**

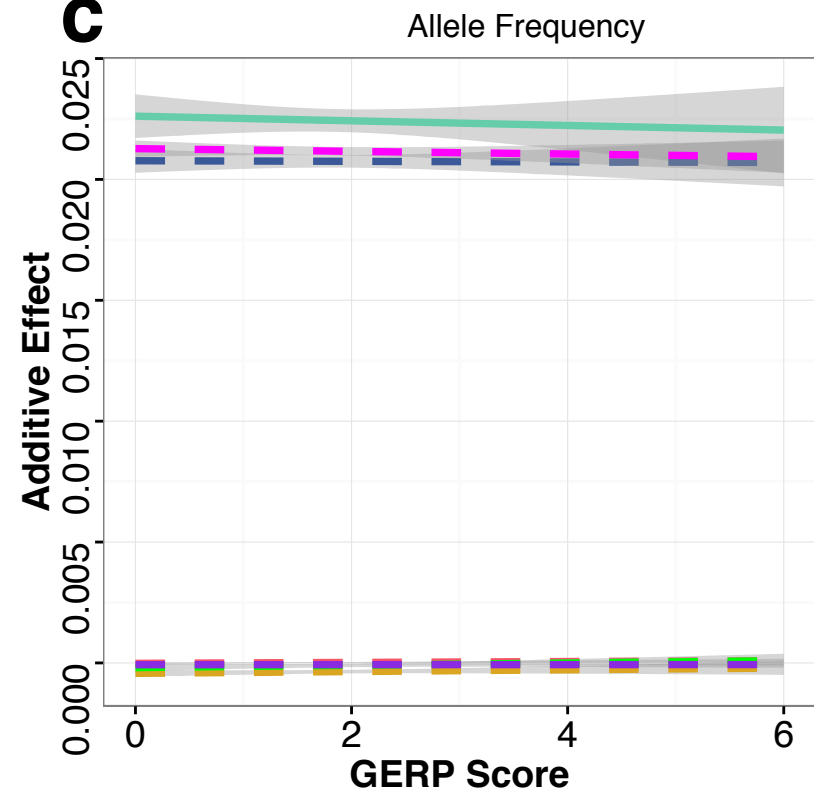

**d**

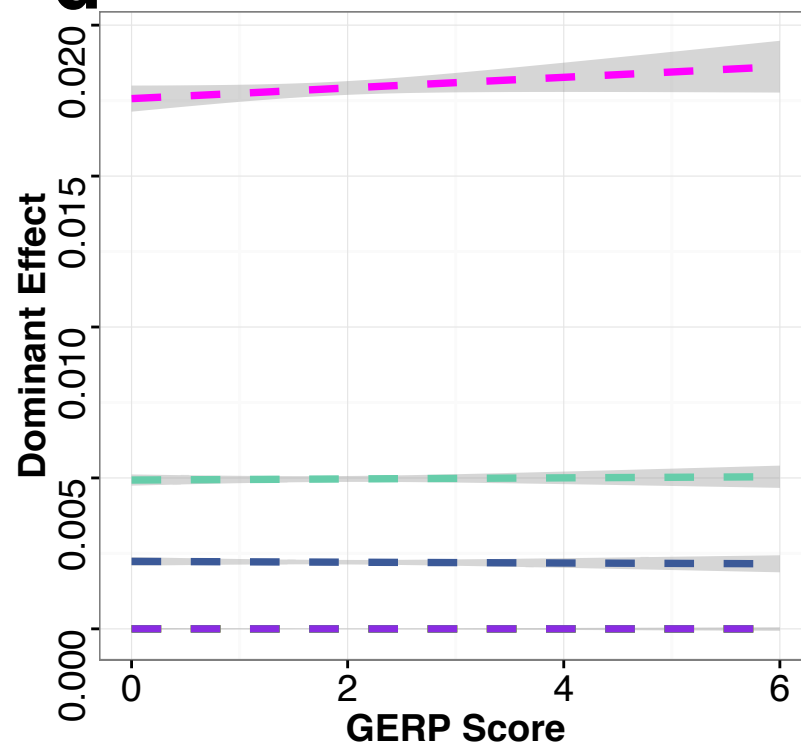

**e**

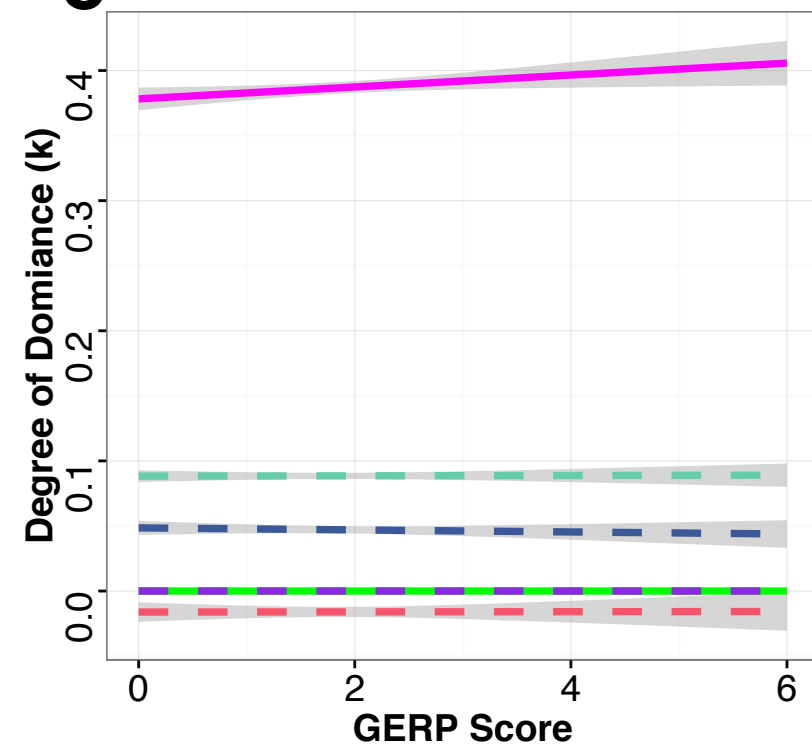

Supplement: S10 Fig — (a) Total per-SNP variance explained for grain yield per se by deleterious (red lines) and randomly sampled SNPs (grey beanplots). (b) Density plots of the degree of dominance (k). Extreme values of k were truncated at 2 and -2 for visualization. (c-e) Linear regressions of additive effects (c), dominance effects (d), and degree of dominance (e) of seven traits per se against SNP GERP scores. Colors in (c-e) are the same as the legend for (b). Solid and dashed lines represent significant and nonsignificant linear regressions, with grey bands representing 95% confidence intervals. Data are only shown for deleterious alleles that explain more variance than the genome-wide mean. (PDF) [file pgen.1007019.s010.pdf]

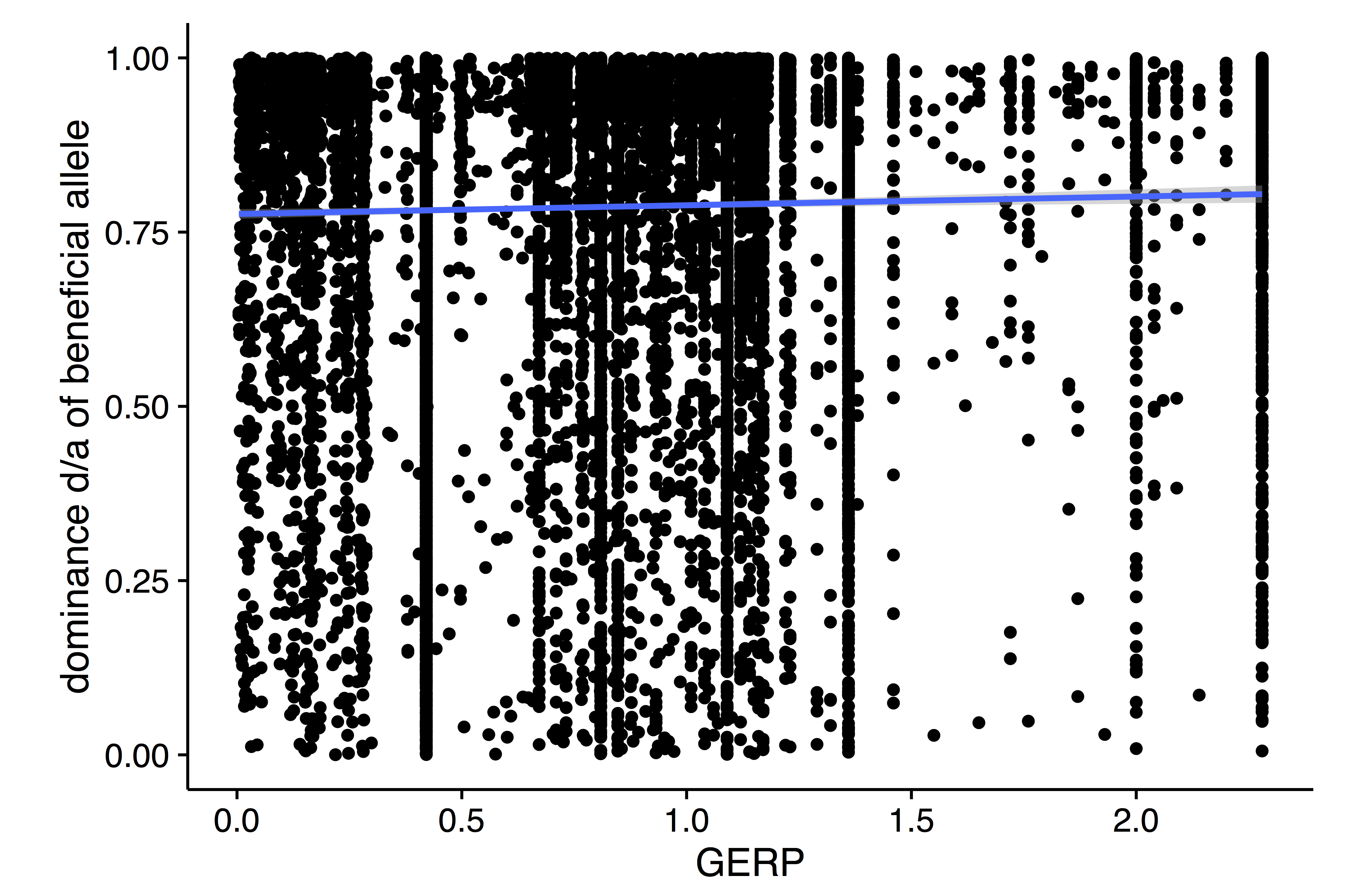

Supplement: S11 Fig — The solid blue line indicates the regression line fitted to data simulated under mutation-selection balance (see Methods for details). (PNG) [file pgen.1007019.s011.png]

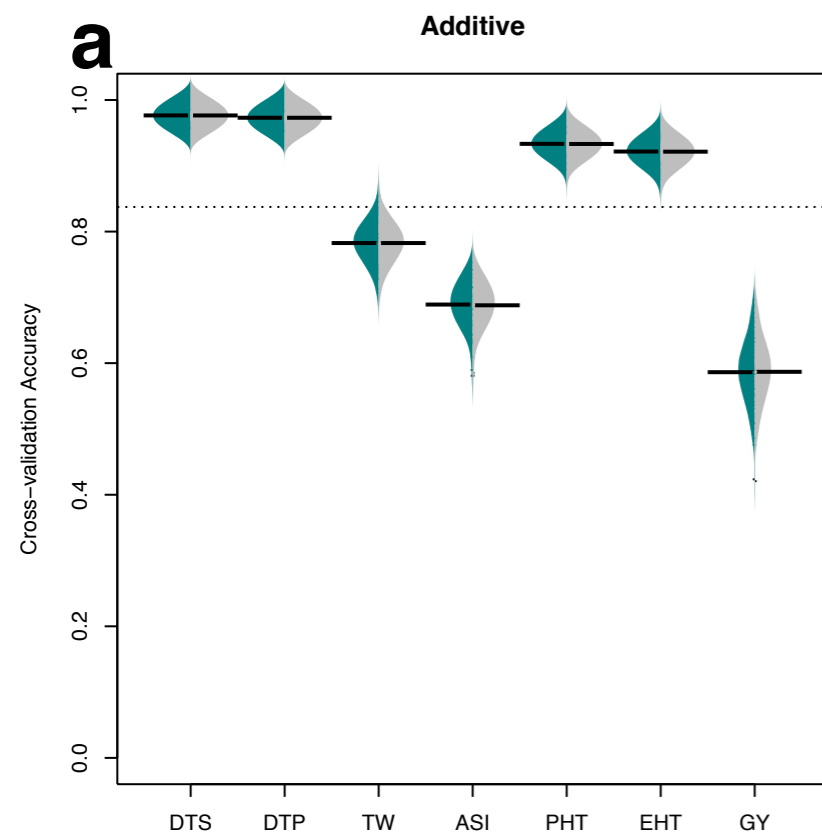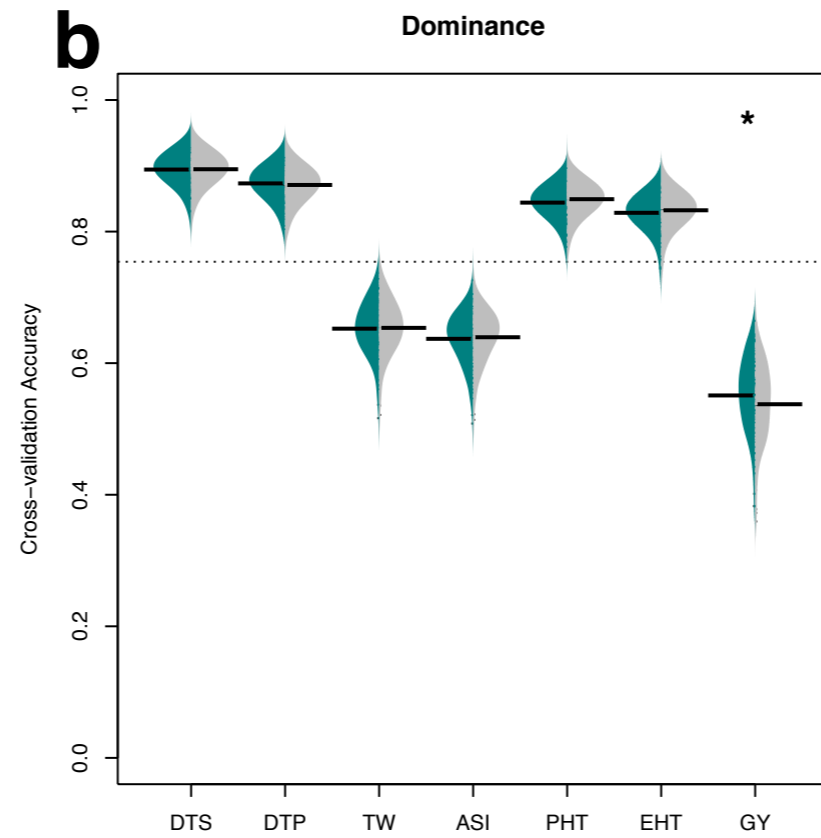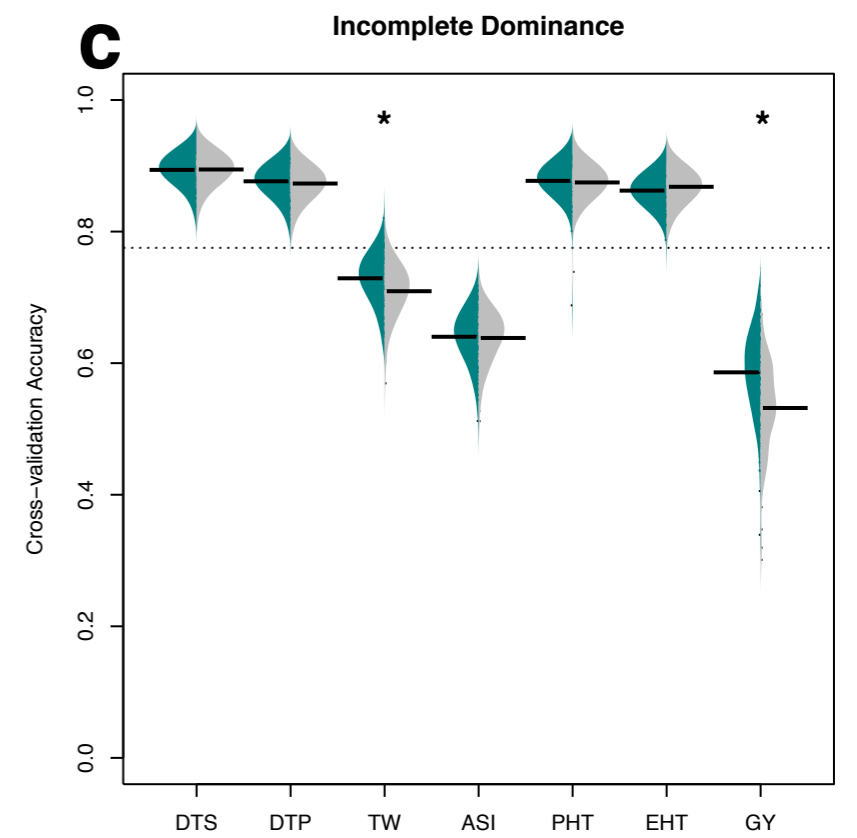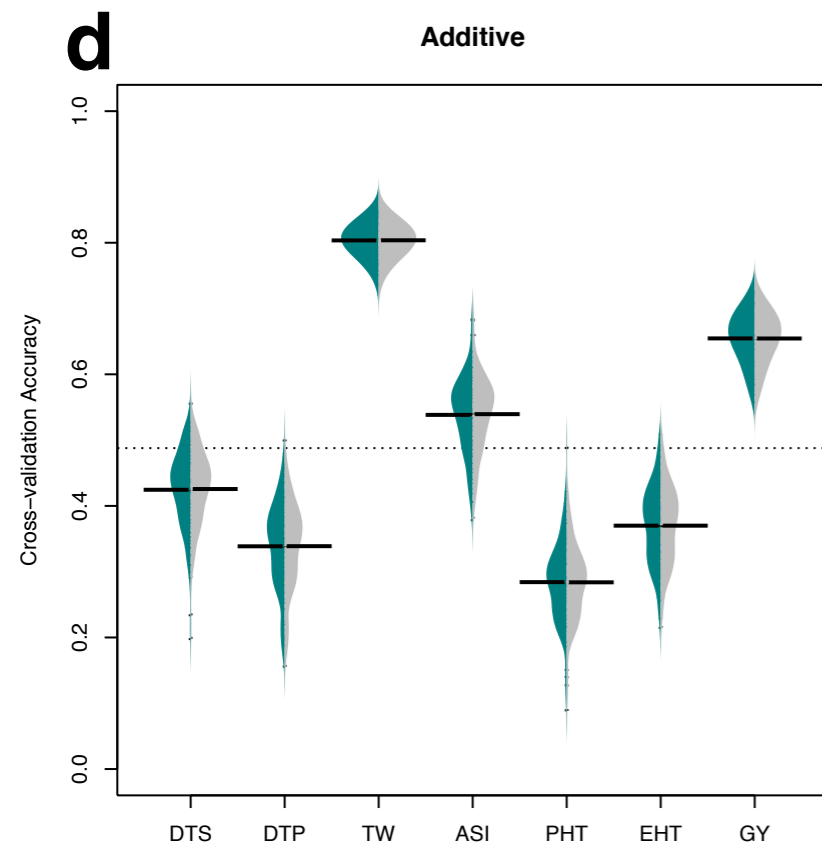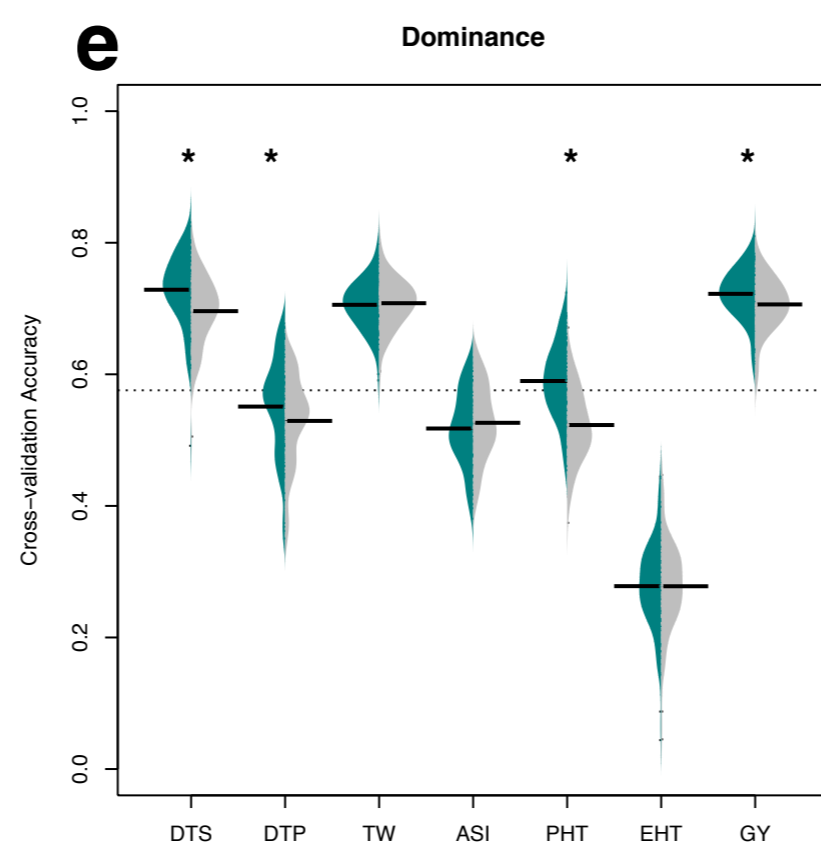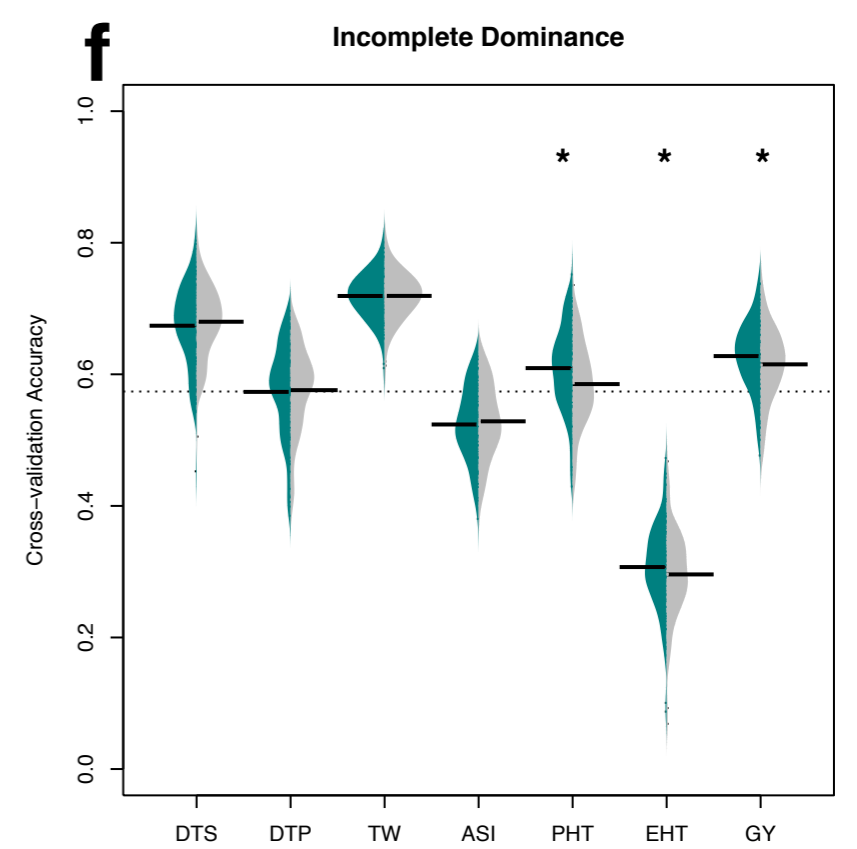

Supplement: S12 Fig — Beanplots represent prediction accuracy estimated from cross-validation experiments for traits per se (a, b, c) and heterosis (d, e, f) under additive (a, d), dominance (b, e), and incomplete dominance (c, f) models. Prediction accuracy using real data is shown on the left (green) and permutation results on the right (grey). Horizontal bars indicate mean accuracy and the grey dashed lines indicate the overall mean accuracy. Stars indicate real data having significantly (t-test P value < 0.05) higher cross-validation accuracy than permuted data. (PDF) [file pgen.1007019.s012.pdf]

# Grain Yield

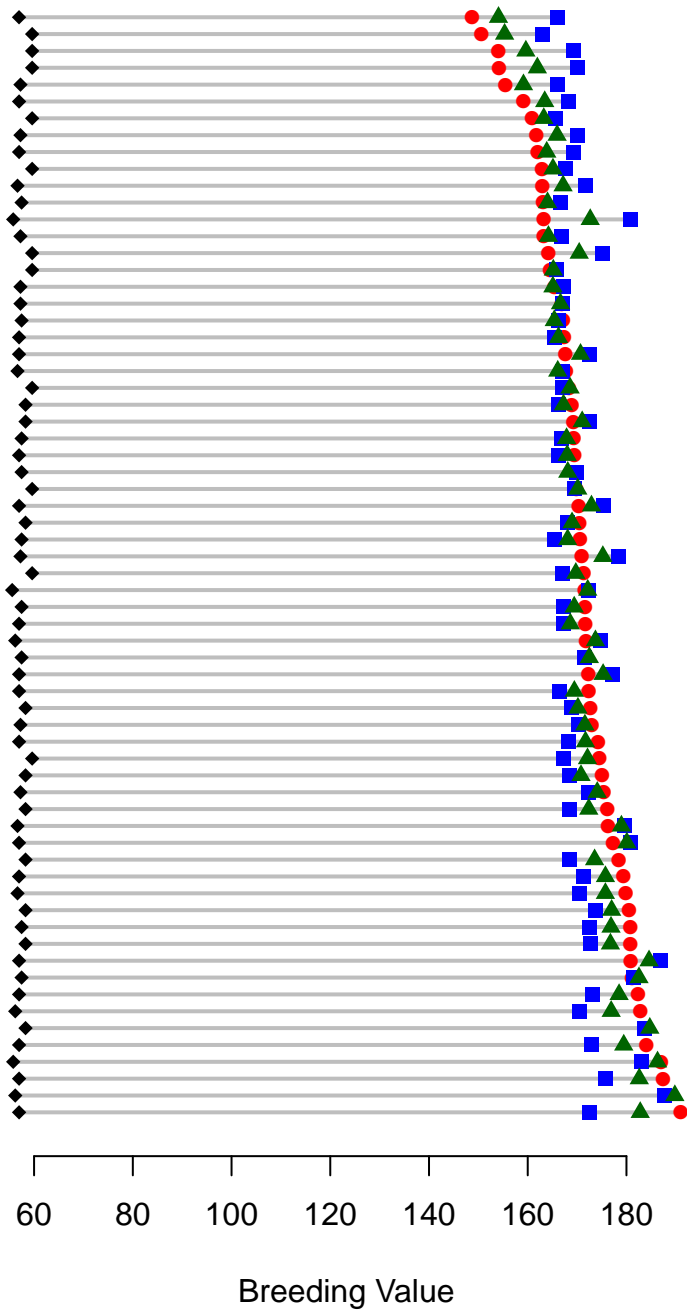

Supplement: S14 Fig — Each line represents the posterior breeding values of a diploid hybrid (red circle), its best parent (black diamond), and predicted breeding values of simulated AAB triploid (blue square) and ABB triploid (green triangle) plants based on estimated effect sizes and dominance values for each SNP. (PDF) [file pgen.1007019.s014.pdf]
