# Supplementary material for: Incomplete dominance of deleterious alleles contributes substantially to trait variation and heterosis in maize
Source: PLoS Genet. 2017 Sep 27;13(9):e1007019. doi: 10.1371/journal.pgen.1007019 (PMC5633198; doi:10.1371/journal.pgen.1007019)
Supplement: S13 Fig — Beanplots represent prediction accuracy estimated from cross-validation experiments for traits per se (a, b) and heterosis (c, d) under additive (a, c) and dominance (b, d) models. Prediction accuracy using real data is shown on the left (red) and permutation results on the right (grey). Horizontal bars indicate mean accuracy of each trait and the grey dashed lines indicate the mean accuracy of all traits. Stars indicate real data having significantly (t-test P value < 0.05) higher cross-validation accuracy than permuted data. (PDF) [file pgen.1007019.s013.pdf]

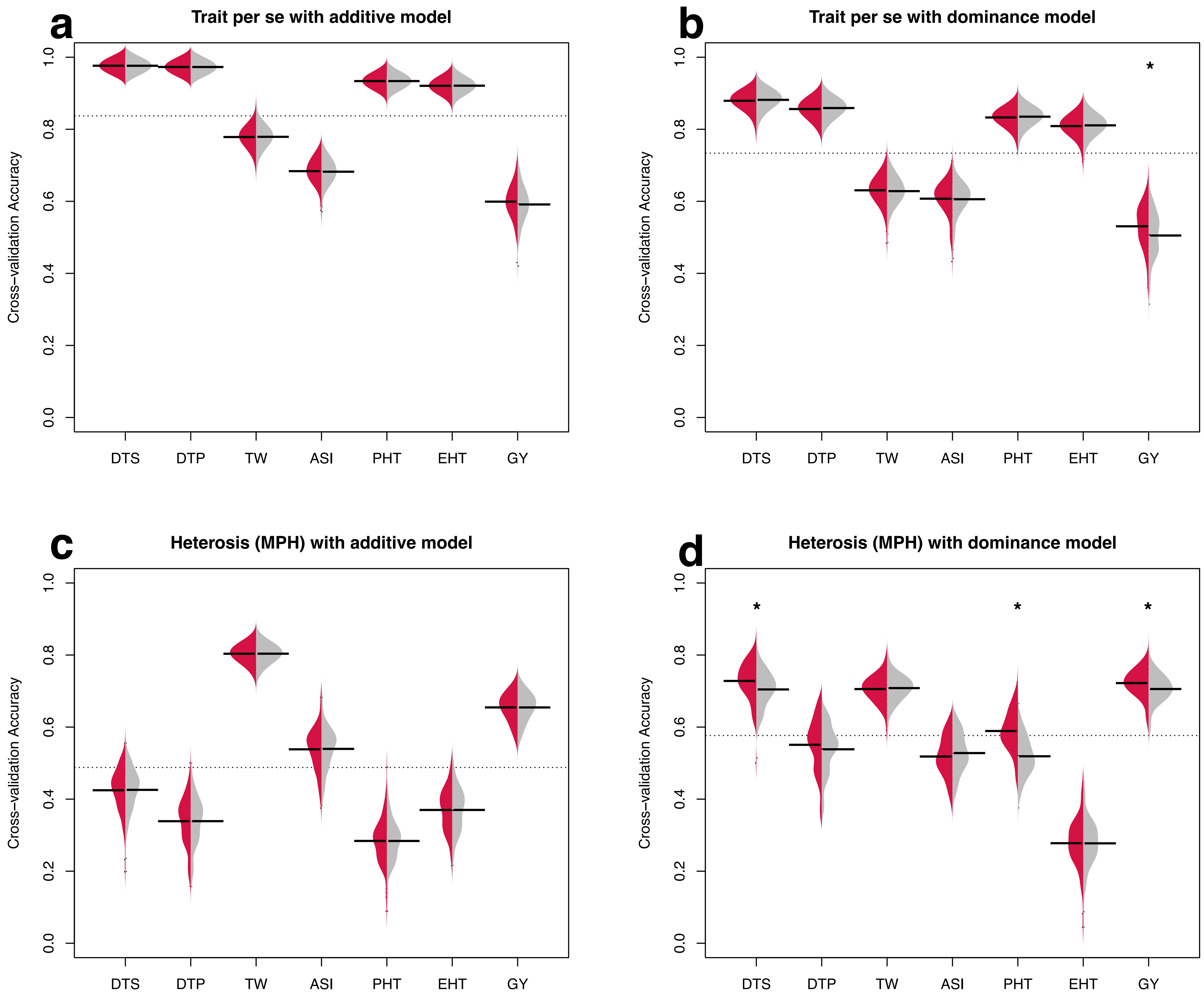
a
Trait per se with additive model
b
Trait per se with dominance model
c
Heterosis (MPH) with additive model
d
Heterosis (MPH) with dominance model
Cross−validation Accuracy
0.0
0.2
0.4
0.6
0.8
1.0
DTS
DTP
TW
ASI
PHT
EHT
GY
*
